# Supersaturation with water explains the unusual adhesion of aggregate glue in the webs of the moth-specialist spider, *Cyrtarachne akirai*

**DOI:** 10.1098/rsos.181296

**Published:** 2018-11-07

**Authors:** Candido Diaz, Akio Tanikawa, Tadashi Miyashita, Gaurav Amarpuri, Dharamdeep Jain, Ali Dhinojwala, Todd A. Blackledge

**Affiliations:** 1 Department of Biology, The University of Akron, Akron, OH 44325, USA; 2 Department of Polymer Science, The University of Akron, Akron, OH 44325, USA; 3 School of Agriculture and Life Sciences, The University of Tokyo, Bunkyo-ku, Tokyo, Japan

**Keywords:** spider silk, aggregate glue, adhesion, *Cyrtarachne*, viscosity

## Abstract

Orb webs produced by araneoid spiders depend upon aggregate glue-coated capture threads to retain their prey. Moths are challenging prey for most spiders because their scales detach and contaminate the glue droplets, significantly decreasing adhesion. *Cyrtarachne* are moth-specialist orb-weaving spiders whose capture threads adhere well to moths. We compare the adhesive properties and chemistry of *Cyrtarachne* aggregate glue to other orb-weaving spiders to test hypotheses about their structure, chemistry and performance that could explain the strength of *Cyrtarachne* glue. We show that the unusually large glue droplets on *Cyrtarachne* capture threads make them approximately 8 times more adhesive on glass substrate than capture threads from typical orb-weaving species, but *Cyrtarachne* adhesion is similar to that of other species after normalization by glue volume. Glue viscosity reversibly changes over 1000-fold in response to atmospheric humidity, and the adhesive strength of many species of orb spiders is maximized at a viscosity of approximately 10^5^–10^6^ cst where the contributions of spreading and bulk cohesion are optimized. By contrast, viscosity of *Cyrtarachne* aggregate glue droplets is approximately 1000 times lower at maximum adhesive humidity, likely facilitating rapid spreading across moth scales. Water uptake by glue droplets is controlled, in part, by hygroscopic low molecular weight compounds. NMR showed evidence that *Cyrtarachne* glue contains a variety of unknown low molecular weight compounds. These compounds may help explain how *Cyrtarachne* produces such exceptionally large and low viscosity glue droplets, and also why these glue droplets rapidly lose water volume after brief ageing or exposure to even slightly dry (e.g. < 80% RH) conditions, permanently reducing their adhesion. We hypothesize that the combination of large glue droplet size and low viscosity helps *Cyrtarachne* glue to penetrate the gaps between moth scales.

## 1. Introduction

Orb webs produced by araneoid spiders depend upon glue-coated capture threads to catch their flying insect prey in mid-flight [1–5]. The aggregate glue droplets form around an underlying flagelliform silk fibre and are composed of a mixture of adhesive glycoproteins surrounded by an aqueous solution of low molecular mass compounds (LMMCs) that include inorganic salts, lipids and a large proportion of small peptides [6]. This combination of chemicals results in a glue droplet that responds rapidly to humidity by reversibly absorbing water from the air and creates a system that readily flows while remaining firmly anchored to the capture silk. Within aggregate glue droplets, the LMMCs of the silk help to expand the droplet as water is absorbed from the atmosphere. This lowers glue viscosity, making the glue spread more readily after coming into contact with a substrate [5,7,8]. Aggregate glue adhesion strength varies with humidity almost tenfold in some species due to changes in viscosity as the droplets absorb and release water to the atmosphere [8].

Surface wetting is important for proper adhesion with larger droplets having the potential to spread further, establishing higher contact area with the substrate and ultimately increasing adhesion strength [7,9]. As humidity decreases, aggregate glue becomes less fluid and is unable to flow into any cracks or grooves present on the substrate, resulting in lower adhesive strength [9]. Lower viscosity allows aggregate glue to spread rapidly across surfaces and increases total contact area.

Prey retention by orb webs is challenging as the gravitational forces and struggling by insects allow many prey to rapidly escape from webs before the spiders can capture them [2,10,11]. Moths are especially elusive to spiders because moth scales flake off and cover glue droplets, which lowers the web's total adhesion to the prey body allowing the moths to escape [1,5,11–14]. But moths are abundant in most ecosystems and one lineage of orb-weaving spiders, the Cyrtarachninae within the large orb-weaving family Araneidae, are moth-specialists whose glue sticks unusually well to moths [1,12,13]. This group includes both the enigmatic bolas spiders, which use prey pheromone mimicry and highly modified bolas webs, consisting of only single threads tipped with large glue droplets, to target-specific species of moths, and genera like *Cyrtarachne* that spin reduced horizontal orb webs and capture a broad range of moth species without using chemical mimicry. At present only a single paper exists on the material properties of *Cyrtarachne* capture threads [15]. In addition to demonstrating the potential for evolutionary adaptation in predatory behaviours, the system is analogous to the challenges faced by synthetic adhesives sticking to contaminated surfaces where dirt particles create a barrier between the adhesive and the substrate [5,9,12,16]. Therefore, studying the material properties of *Cyrtarachne* silk could give insight into how to design synthetic adhesives that can stick well to dirty surfaces.

*Cyrtarachne* are found throughout Asia and Oceania and produce a reduced horizontal orb web with only a few widely spaced capture threads [1,12,17]. This web shape is counter-intuitive for webs specialized on ‘dirty’ prey like moths because a narrower distance between capture threads would result in more strands of capture silk contacting prey, increasing overall adhesion. Indeed, ladder webs are an alternative specialization for moth capture, where large numbers of capture threads gradually wear away the protective coating of scales [1,12]. Instead, *Cyrtarachne* webs usually stick to moths using only single capture threads that are coated with notably large glue droplets [12,13,16]. The reduced length of capture threads in *Cyrtarachne* webs is thus offset somewhat by an increase in the volume of aggregate glue on capture threads [17]. The reliance on single threads to stick to prey suggests that the mechanical properties of *Cyrtarachne* glue droplets are extremely important in retention of moths. Thus, we characterize the adhesion of *Cyrtarachne* capture silk at 50%, 70% and 90% RH, as previously measured, and compare it to several typical orb-weaving species, most often *Larinioides cornutus* [8].

*Cyrtarachne* produce significantly larger glue droplets than other species so that their silk may be stickier simply because it has a lot of adhesive material [13,17]. Indeed, the exceptionally large single glue droplets used by bolas spiders are assembled as the spiders push multiple droplets of glue together [18]. If so, the size of *Cyrtarachne* aggregate glue droplets would be significantly larger than other species even at low humidity where the water has been mostly removed, leaving only the glycoproteins and LMMCs. We therefore compare the volume of fresh glue droplets at 90% RH to droplets dehydrated at 20% RH to determine if *Cyrtarachne* glue droplets simply contain more aggregate silk glue than other species.

Alternatively, the larger droplet size may result from a higher proportion of water in the glue. Extra water could improve adhesion by increasing the droplet volume and thereby increasing the total surface area over which the glue spreads [7]. Increased water content would also decrease viscosity, which would help *Cyrtarachne* glue to spread rapidly across moths and penetrate underneath their scales [12,13,16]. We therefore compare droplet spreading behaviour of *Cyrtarachne* glue to that of several previously investigated species of orb spiders [8]. We also measure the proportion of glue droplet mass composed by LMMCs, glycoproteins and water.

In contrast to those of other orb spider species, *Cyrtarachne* glue droplets rapidly lose adhesion even under humid conditions [17]. Variation within the LMMCs, glycoproteins and/or water present in these droplets might result in silk with time-dependent viscosity whose adhesion strength changes over time. Rapid water loss could result in changes in viscosity that allow the glue to spread initially but then harden to better resist pull-off, as is common in many synthetic adhesives. We therefore compare the performance and hygroscopic properties of fresh and aged aggregate silk, as well as its change in water content.

Finally, the composition of LMMCs in glue droplets plays a key role in both determining glue hygroscopicity and solvating the adhesive glycoproteins [19]. We therefore use nuclear magnetic resonance (NMR) to identify the water-soluble LMMCs present in *Cyrtarachne* aggregate glue and compare them to other orb-weaver species.

Here, we test specializations of spider aggregate glue that may aid in adhesion to ‘dirty’ surfaces such as moth prey, in part to consider what properties are important in the creation of our own synthetic adhesives to stick to ‘dirty’ substrates.

## 2. Methods

### 2.1. Collection of specimens

Nine mature female *Cyrtarachne akirai* were collected from tall grass surrounding several rice paddy fields in Chiba prefecture, Japan [20]. Collection occurred five times between June and October 2015 at the gps location of (35.65635° N, 140.2425° E). *Cyrtarachne* were collected into plastic tubes and then immediately shipped to Akron, Ohio (USA) where they were housed in the laboratory for two to 12 weeks between June and December.

*Cyrtarachne* glue was compared to several ‘typical’ orb-weaving species of spiders, in particular *Larinioides cornutus* whose glue behaviour and chemistry have been intensively investigated. These spiders were collected from Akron, OH as needed and webs were spun in cages as previously described [21].

### 2.2. Housing of *Cyrtarachne*

Spiders were housed in one of three sizes of plastic cages: height × length × width, 27 cm × 17 cm × 16 cm, 20 cm × 20 cm × 20 cm or 37 cm × 24 cm × 21 cm. *Cyrtarachne* require high humidity environments of approximately 90% RH to induce web spinning [22,23]. Cages were designed similar to those used to house *Pasilobus*, a sister genus that also builds similar webs only at high humidity [22]. The floor of each tank was layered with damp towels and filled with approximately 8 cm of water. Tanks were sprayed with water nightly and allowed to dry in the morning. Finally, tanks were wrapped in moist towels during the night to help further maintain adequate humidity. *Cyrtarachne akirai* were fed by placing crickets in their webs, once every seven days. Spiders that did not produce webs were unable to be fed. Capture thread samples were only collected from spiders that had eaten within the last week.

### 2.3. Collection of web samples

Spider tanks were checked for fresh web construction every 3–4 h between 08.00 and 07.00. Fresh webs were most often found between 05.00 and 06.00. Initial time in captivity prior to web making varied between 2 and 14 days and individual spiders built as many as five webs during the experiment. Once a web was constructed, two adjacent samples of viscid thread were collected from the same capture thread. One sample was adhered to a paper card across a 12.6 mm gap for adhesion testing, while the other was collected onto a glass microscope slide; both samples were used for microscopy. Between one and four paired samples were collected from each web, depending on the size of the web and how quickly the spider began to recycle its web once disturbed. Strands were glued to the cards using polyvinyl acetate, which dries relatively quickly but does not dehydrate the silk strand. Samples that could not be tested immediately after collection were stored in microscope slide boxes until use at room humidity, approximately 40%, at approximately 21°C for 30–45 days. These samples make up the ‘one month aged’ condition for comparison in adhesive tests.

The difficulty of collecting silk, the tendency of spiders to recycle their webs and the relative rarity with which spiders constructed webs limited the total number of samples that we obtained. Thus sample sizes represent individual threads pooled across all spiders rather than individual webs.

Similar protocols were used to collect silk samples from comparison species, using webs freshly spun within the last 24 h.

### 2.4. Glue morphology

Suspended glue droplets were photographed at 10× under a Leica DM LB2 optical microscope using an Q-Color5 camera in order to measure average droplet diameters and observe droplet morphology. Photos were analysed using ImageJ to estimate the volume of droplets (Rasband, W.S., ImageJ, US National Institutes of Health, Bethesda, Maryland, USA, https://imagej.nih.gov/ij/, 1997–2018). Droplet radius was measured along each plane and was used to estimate the volume of the droplet using the equation proposed in Liao *et al.* [24]. Some samples were adhered to glass slides and imaged using a scanning electron microscope (SEM) to examine their morphology. These samples were sputter-coated in gold for 1 min and photographed under a Quanta 200 ESEM (FEI Company Inc., Hillsboro, OR), at magnifications ranging from 60 × to 682 ×. Samples were attached fresh and within specimen tanks at room temperature, approximately 21°C.

To study the distribution of glycoproteins within the spread glue droplets of *Cyrtarachne* (*N* = 3), fresh droplets were brought into contact with a glass slide and then stained with periodic acid−Schiff (PAS) (Sigma-Aldrich, Inc. Merck KgaA, Darmstadt, Germany), which selectively stains glycoproteins. The slides were dipped in periodic acid solution for 5 min at 25°C followed by rinsing in distilled water. The slides were then immersed in the Schiff's reagent for 15 min at 25°C. Then the slides were washed in running tap water for 5 min. The slides were counterstained in haematoxylin solution Gill No. 3 for 90 s and then again washed with tap water. Finally, the samples were dehydrated and mounted to be observed and photographed as detailed above. This process removes water-soluble components while leaving the glycoproteins attached to the substrate [25]. Qualitative comparisons were made to the morphology of fresh and adhered *Larinioides cornutus* silk.

### 2.5. Droplet swelling/contraction in response to humidity

Aggregate glue droplets swell and shrink as hygroscopic LMMCs absorb atmospheric humidity [7]. We hypothesized that *C. akirai* glue droplets are supersaturated with water by the spider so that immediately after spinning the droplets contain more water than at equilibrium, even at high humidity, so that droplet volume would permanently decrease over time. To test this hypothesis, 5 mm *C. akirai* threads (*N* = 4) were collected on glass forks and then placed into a custom humidity chamber under an Olympus BX53 light microscope [8]. Humidity was controlled by altering the ratio of dry nitrogen and nitrogen bubbled through a water column. The humidity was initially set to 96 ± 3% RH. Two to three droplets were in view at 20 × magnification and were photographed as humidity was decreased from 96% to 64 ± 3%, then to 43.3 ± 4% and finally to 22 ± 2% RH. Then the droplets were again photographed as humidity increased back approximately to 40%, 60% and finally 90% with 1 min between each step. Percentage change between initial volume at 90% RH and each humidity increment was calculated. Percentage change was averaged for all droplets so that all droplets on a single thread served as a single data point.

To better understand the effect of time *per se* rather than humidity on droplet volume, three additional strands of fresh *C. akirai* capture silk were collected as described above and placed in the chamber at 100% RH. The droplets were photographed at time points 0, 5 min, 10 min, 15 min, 30 min, 1 h and each subsequent hour until 6 h of age. The humidity was kept constant for the entirety of the experiment. Droplet volume was calculated at each time point and then the slope of this set was calculated to determine the rate of water loss as a function of time.

### 2.6. Aggregate glue adhesion strength

Adhesion tests were conducted using a Nano Bionix^®^ tensile tester (MTS Systems Corp., Oak Ridge, TN, USA). Capture threads were tested on smooth square glass plates measuring 5 mm × 5 mm that were cleaned between subsequent tests using isopropyl alcohol [8]. The 12.6 mm length threads were preloaded onto a glass substrate until they registered 15 µN, held in place for 6 s, and were then pulled from the substrate at 0.1 mm s^−1^, until failure [26]. The speed and thread length were chosen to coincide with previous studies [8]. For the ‘fresh’ test condition, push–pull tests were conducted at approximately 90% RH (*N* = 24). Samples were collected from 17 webs and tested within a plastic humidity chamber where RH was maintained through a combination of N_2_ gas bubbled into the chamber through a column of water and humidifiers in the surrounding room. Force as a function of time was measured by the Nano Bionix machine until the glue completely detached from the surface. Peak forces were noted and total work to release was calculated from the force–displacement curves.

Aggregate glue from most species of spiders maintains its adhesion as long as the atmospheric RH is high enough and adhesion of aged samples can be recovered through rehydration [27]. However, it was reported that *C. akirai* aggregate glue lost its adhesion within a few hours even under high humidity conditions [17]. We therefore tested additional samples of capture thread that were allowed to age at room humidity for one month prior to testing. These rehydrated adhesion tests were conducted at 90% RH (*N* = 12) with exposure to 90% RH for 5 min prior to testing. The rehydration time for our one-month tests was significantly shorter than in Opell & Schwend [27]. Therefore, we measured additional seven-month-old samples that were kept at 90% RH like in Opell & Schwend [27]. The samples were then tested as previously described at 90% RH (*N* = 8).

Peak force and total work to release were calculated for each test condition along with standard errors. *t*-Tests assuming unequal variance were calculated for both peak force and work to release (fresh, aged—short rehydration, aged—long rehydration), using JMP Pro^®^, v.12. SAS Institute Inc., Cary, NC, 1989–2007.

We compared the droplet adhesion strength of fresh *Cyrtarachne* glue to several previously investigated species of orb spiders at 50%, 70% and 90% RH [8]. We then normalized this adhesive strength by the average volume of aggregate glue/mm (measured at 40% RH) for *Cyrtarachne* and compared these to previously measured species to determine if the maximum strength of *Cyrtarachne* glue droplets is exceptional per volume [8]. We paid special attention to adhesion strength of each species at their best RH [8].

### 2.7. Glue spreading rate

To estimate glue viscosity, we compared the spreading rate of *C. akirai* glue to the aggregate glue produced by other orb-weaving spiders [8] and also between aged and fresh glue. The 5 mm silk threads were held on a glass fork and inserted into a custom-made humidity chamber mounted on an Olympus BX53 microscope and imaged using a Photron FASTCAM SA3 high-speed camera at 1000 frames per second (fps). N_2_ gas was pumped through water and into the chamber to reach 90% humidity [8]. Silk strands were given 5 min to acclimate to the new humidity. The spider silk thread was then brought into contact with a glass substrate using a motor-controlled manipulator. The speed of approach was 0.1 mm s^−1^. The transparent glass substrates had been cleaned by sonicating for 15 min each in chloroform and acetone, followed by drying in N_2_ gas [8]. Two droplets were chosen from each video depending on video clarity and analysed using the ProAnalyst motion analysis software (Xcitex, Cambridge, MA). Owing to limitations in viewing angle, it was not possible to measure the rate of spreading when the contact radius was smaller than the original radius of the glue droplet. Tracking began when droplets came in contact with the substrate. Contact was determined by a characteristic colour change as light began to refract through the substrate: droplet layer. Individual coordinate systems were created for each droplet originating at their respective centres. *X*-*Y* coordinates were tracked on the positive *y*-direction edge of the droplet as shown in the electronic supplementary material, appendix a1. The measured coordinates were then translated into a glue droplet radius.

The average spreading ratio as a function of time was plotted. Spreading ratio was defined as the radius of the glue droplet in contact with the surface normalized using initial droplet radius (*r*/*R*). Values were averaged between droplets from the same thread and standard error calculated between thread samples (*N* = 8). Maximum spreading radius was averaged for each spreading condition. Average curves were compared between fresh and aged conditions. Using these *r*/*R* values, we used the concept of shift-factor, validated previously, to calculate the change in viscosity with respect to a reference polymer viscosity, 100 cst PDMS synthetic oil [8]. Comparison is made to published data for four species of typical orb-weaving spiders collected using identical protocols [8].

### 2.8. Capture thread peeling from glass substrates

After their spreading behaviour was recorded as stated above, capture threads were then peeled from the glass substrate and recorded at 60 fps to visualize failure mechanisms [8]. Owing to the long time frame of peeling tests, this lower fps was chosen to maximize video length to video overall droplet peeling behaviour. The speed of detachment was 0.1 mm s^−1^.

### 2.9. Capture thread composition

The rapid and irreversible loss of glue droplet volume over short times is unique for *Cyrtarachne*. We hypothesize that the aggregate glue is ‘supersaturated’ with water when it is first spun—having more water than can be sustained at equilibrium with even high humidity. We therefore measured the relative ratio of water to glycoprotein and LMMCs in these glue droplets. Individual fresh capture threads were collected on microscope coverslips and weighed (*N* = 3) to an accuracy of ± 1 μg using a Cahn/Ventron Cahn 21 Automatic Electrobalance (CAHN Ventron, Cerritos, CA). These samples were then allowed to dry overnight in a desiccator to remove free water and reweighed to give the mass of glycoproteins plus LMMCs. Finally, samples were held under deionized water for 5 min to remove the water-soluble LMMCs and then were again dried overnight in a desiccator [25]. Dry samples were then weighed again to give the mass of the glycoproteins and underlying axial thread, allowing us to calculate the percentage mass of water and water-soluble LMMCs, the latter normally comprising 33–66% of the dry weight of the glue droplets [28].

### 2.10. Low molecular mass compounds in aggregate glue

One possible explanation for the unusual behaviour of *C. akirai* glue droplets is a unique chemistry compared to typical orb-weaving spiders. We therefore examined the chemical composition of the water-soluble component of their aggregate glue. While closely related bolas spiders mimic the sex pheromones of their moth prey, these components are not in their silk, and *Cyrtarachne* does not use chemical attractors so that web samples should only contain silk proteins, LMMCs and water [11,15]. The capture silk produced by *C. akirai* was collected by wrapping the whole webs (about 10–15 total webs) on glass pipettes. After the web collection, the silk-laden pipette was washed with deionized water (approx. 10 ml) to remove the water-soluble compounds. The washings were lyophilized for about 12 h to obtain dried water-soluble extract. The extract was refrigerated thereafter until it was used for the solution-state NMR experiments. For the NMR experiments, the extract was dissolved in 1–2 ml of 99.99% deuterated water (Sigma Aldrich) and filled in the 5 mm NMR tube (Norell) followed by loading in the NMR machine for analysis [25]. A sterile pipette was additionally treated in the same way and used as a blank control.

The experiments were carried at 303 K on an INOVA 750 MHz NMR instrument equipped with pulse field gradient (PFG) room temperature probe and Agilent DD2 console setting. For the quantification, proton spin-lattice relaxation (T1) measurements were carried out to determine the suitable recycle delay. The longest relaxation (8 s) was found for the peak at 3.53 ppm (glycine) and accordingly the delay was chosen as 5 * T1 ∼ 40 s for the 1H experiment. The 1D 1H NMR experiment was recorded with 124 scans, 900 pulse width of 9.5 µs, recycle delay of 40 s and an acquisition time of 3.5 s. The results were analysed with the ACD NMR software and peaks were integrated to obtain the relative composition.

## 3. Results

### 3.1. Glue morphology

**Figure 1.**
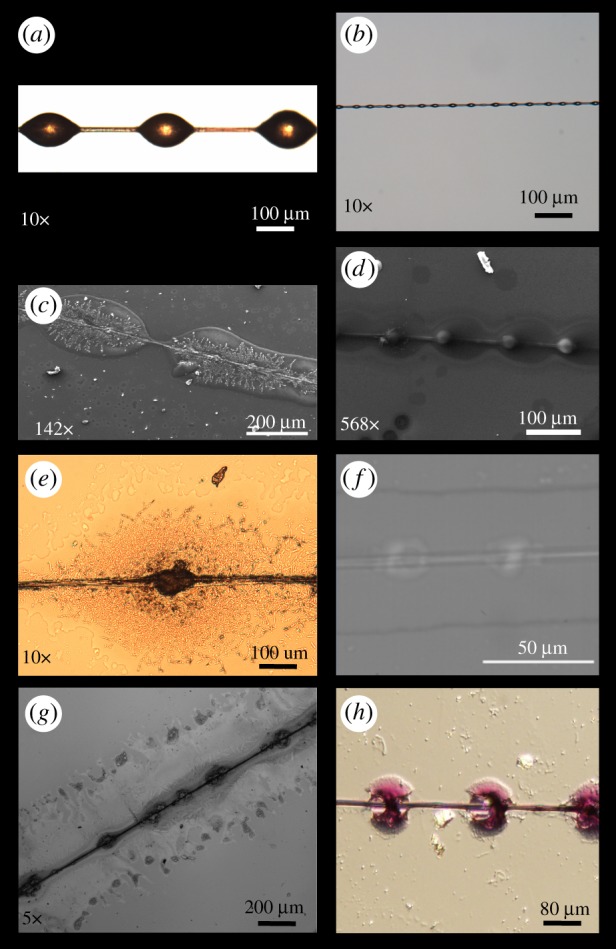
Comparison of *Cyrtarachne* capture threads to those of a typical orb spider. (*a*) Optical image of suspended aggregate glue droplets at 10×. *Cyrtarachne akirai*: glue droplets are large for their size among orb-weavers and can be seen with the naked eye. (*b*) Optical image of suspended aggregate glue droplets from *Larinioides* at 10×: droplets look similar to *Cyrtarachne*, but are significantly smaller. (*c*) SEM image of fresh viscid thread attached to a glass substrate at 90% humidity. The droplet spread like aggregate glue droplets from other species of spiders. However, the glycoprotein spreads further and appears far more defined and crystalline than in other orb weavers, such as *Larinioides* (*d*). Note the well-defined globular glycoprotein core in *Larinioides*. (*e*) Optical Image at 10× of *Cyrtarachne* glue droplet spread on glass. The fibre-like glycoprotein core is visible, like in (*c*), and therefore is not an artefact of our sputter-coating process. (*f*) Optical image of *Larinioides* capture thread spread on glass at 90% RH. (*g*) Optical Image of *Cyrtarachne* capture thread spread on glass with periodic acid–Schiff (PAS) contrast. The sample has been washed and stained in order to better identify the glycoproteins present on the glass. Water-soluble components have been washed off leaving primarily glycoprotein and removing part of the rough topography seen in unwashed silk. The core is clearly visible as observed in other species. The glycoproteins, however, extend to the edge of the droplet edge and are present in large quantities throughout the entire droplet. The glycoproteins appear fibrous like in (*e*). The glycoprotein core can be seen as a distinct granule, while glycoproteins are present in low enough quantities to be not visible surrounding the core with PAS contrast. (*h*) Optical image of *Larinioides* capture thread spread on glass with PAS contrast to highlight glycoprotein presence.

The viscid strands of *Cyrtarachne akirai* resemble those of other orb-weaving spiders in their ‘beads on a string’ arrangement (figure 1*a,b*), but with exceptionally larger aggregate glue droplets [17]. Mean droplet diameter was 151.6 ± 28.2 µm by 99 ± 25.6 µm (mean ± s.e.) at approximately 90% RH for *Cyrtarachne* compared to 32 ± 4 µm by 23.3 ± 2.9 µm for the similarly sized spider *Larinioides cornutus* at approximately 90% RH as reported in Opell *et al.* 2011 [29]. Droplet volumes for samples at room humidity, 40–60% RH, were much smaller than fresh samples at 90% RH (fresh: 832.1 ± 72.8 µm^3^ (*N* = 220), dry: 4.4 × 10^6^ ± 3.4 × 10^4^ µm^3^ (*N* = 84)). This shows that the vast majority of the freshly spun glue droplet, approximately 99.8% of its volume, is composed of water.

The glycoprotein cores of the droplets are easily visible and spread to the outside edges of the droplets when adhered to glass (figure 1*c,d*). In contrast to typical orb spiders, the glycoproteins in *C. akirai* glue appeared almost crystalline after spreading with only a narrow band of clear zone at the edge of the droplet (figure 1*c,d*). The glycoprotein cores of *Larinioides* droplets appeared smoothly spread out from the fibre, without the fibre-like structure seen in *C. akirai* (figure 1*b*). This fibre-like structure could be due to irreversible changes in the structure of the glue proteins. Using PAS contrast, *C. akirai* aggregate glue core was clearly visible as seen in other species. The glycoproteins form rough tributary-like or rivulet**-**like structures [25]. When *Larinioides* capture thread is spread on glass with contrast there are visible glue tributaries and a glycoprotein core as a distinct granule [25]. We found the tributary-like glycoprotein core was consistent throughout our photos for *Cyrtarachne* and is not an artefact of our sputter-coating process (figure 1*c–g*).

### 3.2. Aggregate glue adhesion strength

**Figure 2.**
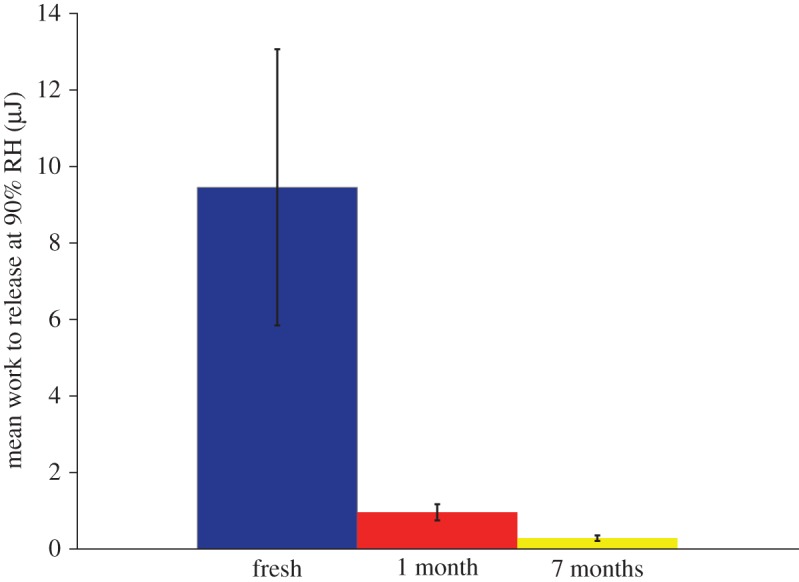
Work to release (μJ) of fresh *Cyrtarachne akirai* capture silk compared to one- and seven-month-aged silk at 90% RH. *Cyrtarachne* capture threads adhere at least 10 times stronger when fresh compared to aged (*p* = 0.2740, 0.0237). Capture silk from other orb spiders maintains adhesion across similarly long time periods.

**Figure 3.**
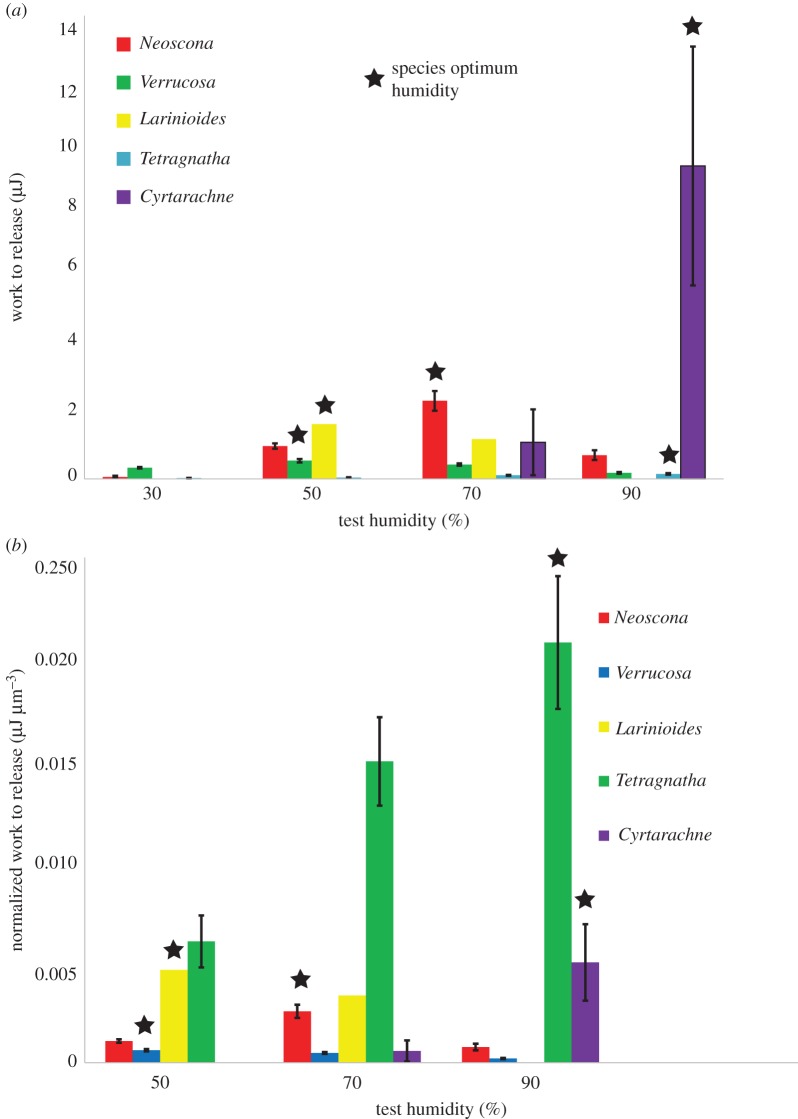
Comparison of work to release for glues from diverse spiders. (*a*) Aggregate glue work to release values during push–pull tests of several commonly studied spider species. *Cyrtarachne*, purple, capture threads require significantly more work to detach from glass substrates than other previously tested spider species, but only at 90% RH (its typical foraging humidity). *Cyrtarachne* glue performance drops rapidly at 70% RH and adhesion tests at 50% RH were so weak that they were unmeasurable. Asterisks show the humidity of maximum adhesion for each species. (*b*) Work to release normalized by average glue volume (measured at 40% RH so that it does not include the extra water incorporated in freshly spun *Cyrtarachne* glue droplets). *Cyrtarachne* aggregate glue adhesion to glass is comparable to other spider species once normalized for its greater initial volume. Data for non-*Cyrtarachne* species taken from Amarpuri *et al.* [8].

Table 1 shows the measured average work to release and peak force before break of 90% RH tests, including fresh, one month aged and seven months aged which were allowed 3 days to acclimate to 90% RH. *C. akirai* capture threads adhere at least ten times stronger when fresh compared to seven month aged (*p* = 0.0237) (figure 2). *C. akirai* glue droplets had zero measurable adhesion at 50% RH and work to release fell substantially at 70% relative to 90% RH (*p* = 0.052). The adhesive strength of *Cyrtarachne* glue droplets was approximately 8 times higher than other species at 90% RH. The greater adhesion strength is largely due to a greater glue volume, which is also approximately 8 times higher (figure 3).

**Table 1.** Work to release and peak force for adhesion tests. *p*-Values are for *t*-tests compared to the fresh condition.

	fresh silk (*N* = 27)	one month aged (*N* = 12)	seven months aged (*N* = 8)
work to release (μJ)	9.46 ± 3.61	0.96 ± 0.21 (*p* = 0.038)	0.29 ± 0.07 (*p* = 0.04)
peak force (μN)	707.82 ± 154	198 ± 22.4 (*p* = 0.013)	94.56 ± 12.1 (*p* = 0.03)

### 3.3. Aggregate glue droplet response to humidity and time

**Figure 4.**
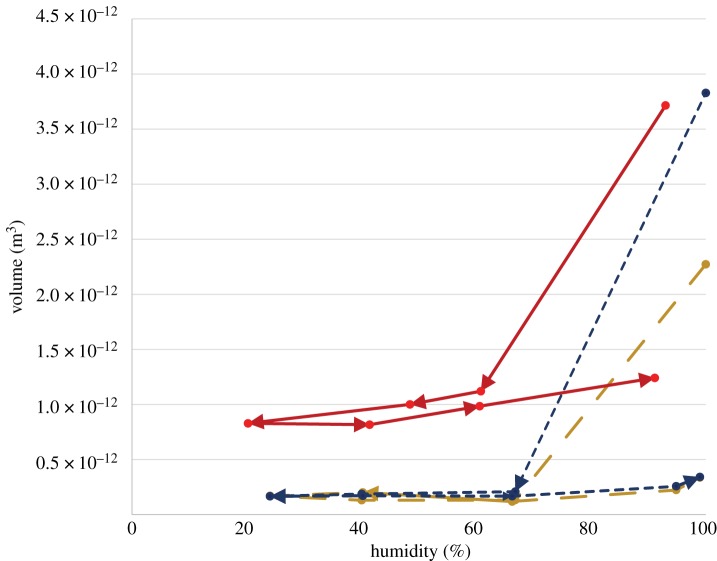
Droplet volume as a function of humidity for fresh *Cyrtarachne* capture threads. Arrows correspond to transitions in humidity as droplets are cycled from high to intermediate to low humidity and back. Droplets lose the majority of their water in the first transition from 90% RH and never return to their original size, even after prolonged exposure to 90%.

**Figure 5.**
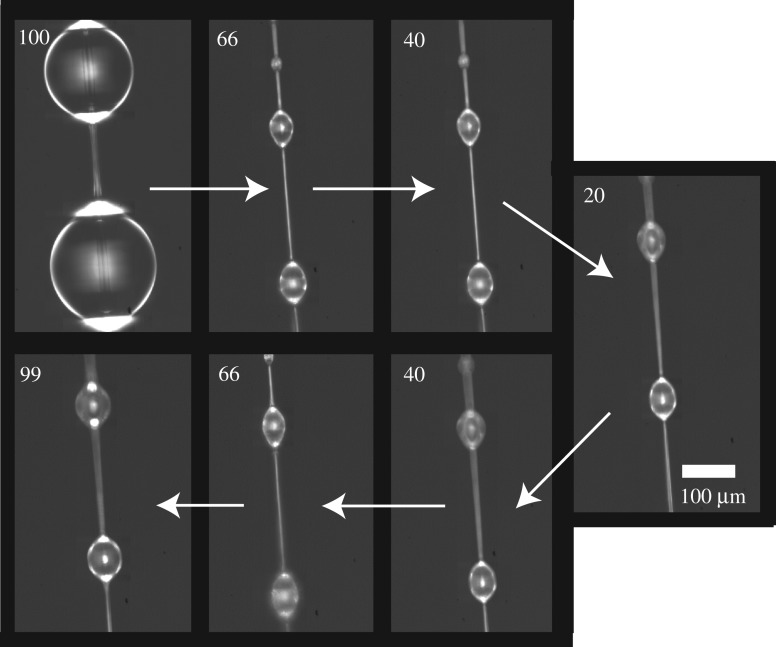
Swelling of a fresh droplet under changing humidity. The initial radius of fresh *Cyrtarachne* aggregate glue droplets at approximately 100% RH is significantly larger than any other condition and is never recovered. Aggregate glue droplets still show a hygroscopic response like other species of orb spiders, reversibly decreasing in size at lower humidity and increasing in size at higher humidity, but the droplets never fully recover their initial radius even with prolonged exposure to 90% humidity.

*Cyrtarachne akirai* droplets are unique in the degree to which they lose water during exposure to low humidity and in the irreversibility of that loss. For fresh viscid threads, droplet volumes were 14 ± 4 times larger at 90% RH compared to 20% humidity (figure 4). By contrast, droplet volume changed only 1.7 times for *Larinioides cornutus* [29]. *Cyrtarachne akirai* droplets never regained their lost water, increasing only 2.4 ± 1.0 times their 20% RH volume after re-exposure to 90% RH (figure 4). While *C. akirai* glue droplets did show reversible uptake of atmospheric water like other orb spiders, its magnitude was small compared to the volume of water that was permanently lost (figure 5).

**Figure 6.**
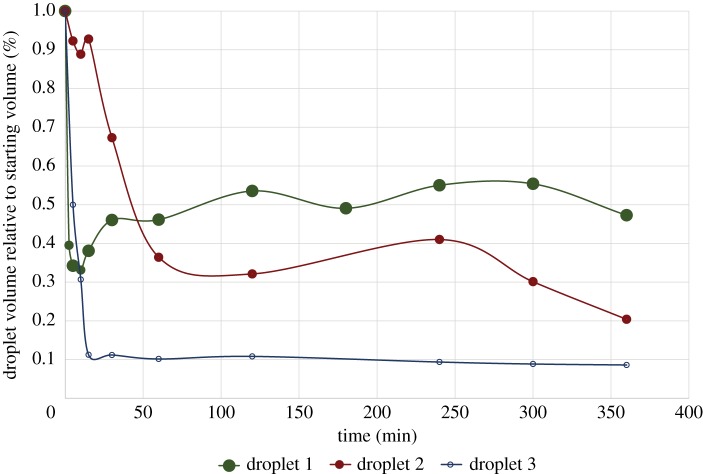
Glue droplet volume loss over time at 99% RH. Droplet volume over time is shown for three glue droplets, denoted by colours. Glue droplets lost 50–90% of their volume within approximately 30 min. This behaviour is unique to *Cyrtarachne* aggregate glue as most orb spiders maintain glue droplet volume under constant humidity.

Water was lost from droplets even without exposure to dry conditions. Droplet volume decreased by as much as 90% within 30–60 min for capture threads maintained between 90 and 99% RH (figure 6).

### 3.4. Spreading and detachment behaviour of aggregate glue on glass

**Figure 7.**
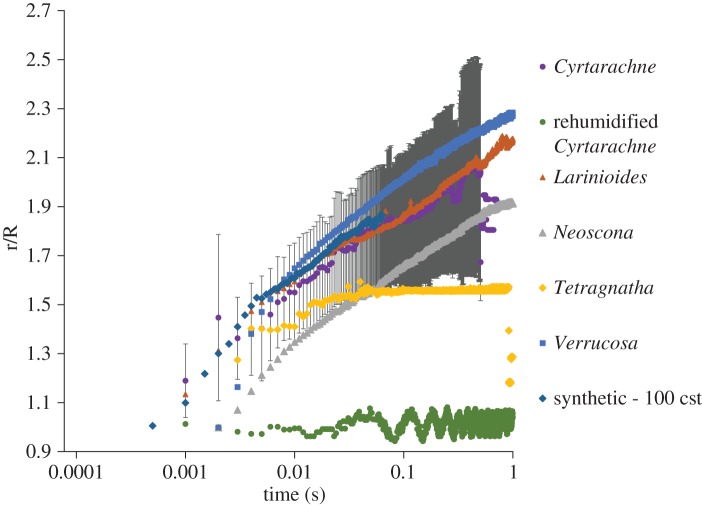
Glue droplet spreading rates at 90% RH. Spreading radius normalized by initial droplet radius for fresh and aged *Cyrtarachne* glue droplets compared to other spiders and a known synthetic control [8]. Old, rehumidified *Cyrtarachne* glue droplets showed zero droplet spread (hence standard deviation is not visible) but shaking of the camera led to slight variation < ± 4%. *Cyrtarachne* glue droplets show similar viscosity at 90% RH to other species of orb spiders in their sub-optimal ‘over lubricated’ states [8].

Fresh glue droplets spread to 2.1 times their initial diameter in 1 s (*N* = 3). This spreading rate corresponded to a viscosity of approximately 100 cst, approximately 1000-fold lower than typical orb spiders at their ‘best’ viscosity for adhesion. Aged droplets failed to spread [8] (figure 7). This means that fresh aggregate glue droplets of *C. akirai* spread upon contact with glass substrates like other orb-weaver species glue droplets do when they are ‘over lubricated’ beyond their optimum humidity for adhesion.

**Figure 8.**
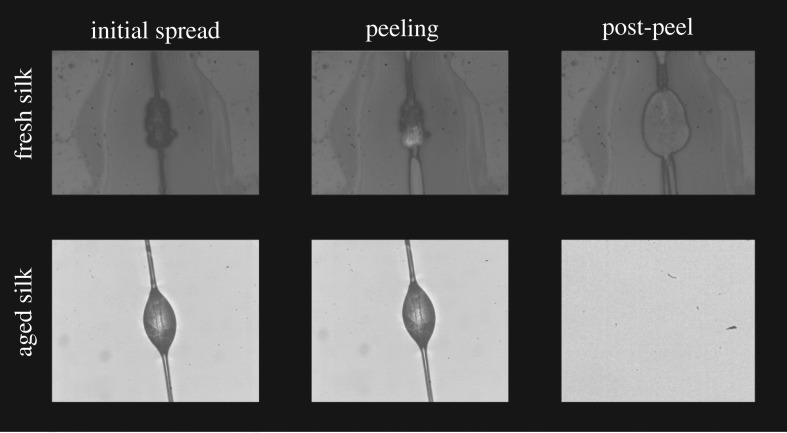
Peeling behaviour of *Cyrtarachne* glue droplets from glass for fresh and aged strands. Fresh aggregate glue droplets of *Cyrtarachne* spread upon contact with glass substrates like other orb-weaver species. The glycoprotein core can be seen in the middle of the droplet. The droplet extends and begins to peel from the outside inward as the capture silk is pulled from the substrate. Post-peeling photos showed the glue residue left on the glass. The capture thread and glycoprotein core are removed from the glass upon adhesive failure but most of the surrounding droplet remains firmly attached. By contrast, aged strand droplets fail to spread upon contact with glass. As such, the droplet retains its initial size and shape with no glycoprotein core visible. As the capture thread is pulled from the substrate, the entire glue droplet rapidly peels as a single unit and there is little to no residue left after the glue droplet is removed from the substrate.

After spreading, the glycoprotein core was still visible in the middle of the droplet. As the capture thread was pulled from the substrate the droplet extended and began to peel from the inside outward. Post-peeling photos showed significant glue residue left on the glass, indicating cohesive failure. The capture thread and core pulled away from the glass upon adhesive failure (figure 8). By contrast, aged droplets failed to spread upon contact with glass. As such, the droplet retained its initial size and shape with no glycoprotein core visible. As the capture thread was pulled from the substrate, the entire glue droplet failed to extend and peeled as a single unit. There was little to no residue left after the glue droplet was removed from the substrate.

### 3.5. Capture thread composition

Water comprised 87 ± 3.6% of the total mass of fresh glue droplets. LMMCs were 28 ± 8.1% of the total dry mass while glycoproteins and the flagelliform thread contributed the remaining 72 ± 8.1% of total dry mass.

**Figure 9.**
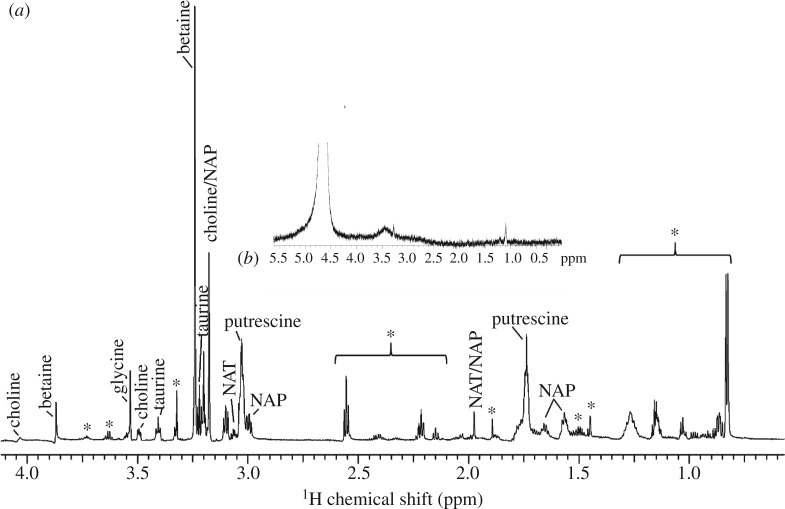
H solution-state NMR of water-soluble components present in the aggregate glue of *Cyrtarachne akirai*. (*a*) Peaks that have been identified as components in the glue of previously studied spider species are labelled. The asterisks mark peaks for unidentified compounds that have largely not been seen in other orb spiders. Inset (*b*) is the spectrum taken from a sterile pipette used as the blank control. NAT, *N*-acetyltaurine; NAP, *N*-acetylputrescine.

The water-soluble compounds of *C. akirai* aggregate glue were analysed and compared to other previously studied species using 1H Solution NMR. The identified low molecular mass organic compounds were hygroscopic and commonly found in the aggregate glues of other species of spiders in different combinations and proportions [19,25,28,30–35]. The peak assignments and identification of these LMMCs were done with respect to the already established data in the literature [19,25,30,34,35]. The key LMMCs detected and their respective approximate relative compositions were putrescine (47%), n-acetyl putrescine (20%), betaine (11%), glycine (7%), taurine (6%), choline (5%) and n-acetyl taurine (4%). A host of peaks were also detected specifically in the chemical shift region (0.5–2.5 ppm), but could not be identified. Some of these unidentified peaks have been seen in the capture glue of *Zygiella atrica,* but they are still not identified [34] (figure 9).

## 4. Discussion

*Cyrtarachne akirai* is a moth-specialist predator whose silk capture threads are able to adhere to moths despite the ‘sacrificial’ layer of scales covering moth wings and bodies [17]. How *Cyrtarachne* silk sticks to moths is unknown, although it has been suggested that the capture silk is significantly stickier than other orb-weavers. *C. akirai* does produce unusually large glue droplets with high adhesive strength, but we found that this adhesion, on glass, was similar to typical orb-weaving spiders when normalized for its larger glue volume [17]. Thus, some other aspect of glue performance may be much more important in how the silk adheres to moths than total adhesive strength *per se*. Particularly, the way in which the glue is able to spread across three-dimensional surfaces might play an important role. *C. akirai* aggregate glue functions at an extremely low viscosity, approximately 1000 times lower than the glue of other species tested at their preferred humidity of maximum adhesion [8]. This low viscosity results from the extremely high water content of the glue, 99.8% by volume for fresh silk compared to less than 69% in other common orb spiders, but is very short lived as the droplets rapidly dry out [36]. The composition of LMMCs in *C. akirai* glue is exceptionally diverse and includes several novel components. These LMMCs may help to explain how *C. akirai* glue can initially supersaturate their glue with water.

The large glue droplets in *C. akirai* capture silk are not simply a function of body size. While we did not measure body size, the *C. akirai* in this study were smaller than or similar in size to the average sizes of previously studied species [8]. Normally, glue volume scales closely with body size across different species of spiders but the increased glue volume of *C. akirai* does not result from this because *C. akirai* is a moderately sized spider; they measure 10–13 mm for fully mature females compared to 6.5 mm–14 mm for *Larinioides cornutus* [37,38]. When normalized by glue droplet volume in this way, the large size of *C. akirai* aggregate glue droplets does not generate disproportionately higher adhesion (electronic supplementary material; figure 3). Instead, we hypothesize that the large volume and low viscosity of the glue droplets may play a key role in allowing the glue to spread across complex three-dimensional surfaces like moth scales.

*Cyrtarachne akirai* glue droplets rapidly lose their ability to spread across surfaces, and hence lose their adhesion. This loss occurs in response to both drying and short-term ageing and is irreversible. The average work to release for fresh silk at 90% RH decreased ten-fold after ageing for one month and it continued to drop with ageing. Seven-months-aged silk showed one-fifth of the work required to detach compared to the one-month-aged silk samples (electronic supplementary material; figure 2). Tests at less than 90% RH lost the majority of their adhesive strength with strands having unmeasurable adhesion below 60% RH, at least partially due to loss of water content. The loss of adhesion in response to drying was so rapid that it likely explains the large variability in fresh 90% humidity tests (electronic supplementary material). Especially for tests early in our experiment, there was enough lag time between collecting the fresh strand and transferring it to the humidity chamber that the glue likely began to desiccate at room humidity. Thus some values underestimated adhesive performance and the distribution of data was left-skewed with the majority of tests falling below the average.

The permanent loss of water by *C. akirai* glue contrasts with previously studied spider silks, which reversibly swell and shrink to repeatable sizes as humidity changes [5,27]. This behaviour is analogous to a hydrogel that swells under humidity but does not allow the water (and in this case suspended glycoproteins) to flow once coming into contact with a substrate. It is possible that the droplets simply do not contain enough LMMCs to keep them hydrated as they comprise a smaller percentage of overall glue droplet dry mass compared to other previously studied species [8] (electronic supplementary material). However, we hypothesize that this water loss causes the irreversible loss of adhesion due to ageing or drying through a phase change occurring within the glycoprotein component of the glue droplets as water concentration is decreased, which causes the partial collapse of glycoproteins within the droplets. This hypothesized phase change is similar to the irreversible collapse of the glue droplets seen in other spiders once the LMMCs are removed, but occurs in *C. akirai* even when LMMCs and humidity are maintained. Alternatively, it is possible that one of the novel LMMC components may cause cross-linking of the glycoproteins over time, reducing spreading. However, the rapid loss of adhesion in *C. akirai* capture silk also occurs in response to drying over very short time periods (electronic supplementary material; figure 7). This supports our hypothesis that supersaturation with water maintains the glycoproteins in a mobile, but unstable state that begins to degrade through irreversible loss of water as soon as the droplets are spun. In other words, *Cyrtarachne* may supersaturate its glue, by actively pumping water into them during spinning of capture thread to achieve large droplet volume and rapid spreading due to low viscosity.

The viscosity of aggregate glue varies 10 000-fold as droplets absorb and release water to the atmosphere, but adhesion is maximized for many different species of spiders at a strikingly similar viscosity of approximately 100 000 cst [8] (electronic supplementary material). *C. akirai* glue showed remarkably lower viscosity of only 100 cst at maximum adhesion compared to other species. However, these spreading rates are similar to *Larinioides* and *Verrucosa* tested at 90% RH (electronic supplementary material; figure 7). At this low viscosity, the glues of most other spiders overspread such that droplets coalesce and adhesion is reduced approximately 2–3-fold as the droplets fail cohesively during detachment (electronic supplementary material). Despite their low viscosity, *C. akirai* glue droplets do not show this overspreading behaviour. The glue droplets spread faster than other species at optimum humidity, and ultimately reach equilibrium at the same relative distance. *Cyrtarachne* may rely on speed of spreading combined with large droplet volumes to overcome moth scales. However, it is important to note that *C. akirai* couples this low viscosity with an ability to remain firmly attached to the flagelliform thread. This contrasts with both the 100 cst polymer solution and the behaviour of the glue of other orb-weaver spiders at similarly low viscosity (when RH is well above their optimal adhesion), which has difficulty maintaining connection to capture threads as its cohesion is extremely low leading to increased occurrences of bulk failure during peeling [8]. These tests serve to show that lower viscosity does not necessarily lead to increased adhesive strength. Thus, *Cyrtarachne* glue droplets must somehow avoid sacrificing high bulk cohesive forces at this low viscosity (the mechanism of which is not yet understood).

The aggregate glues of all currently described orb-weaving spiders (approx. 14 species across three families) are composed of different proportions of a subset of the same 13 organic LMMCs [25,28]. While *C. akirai* glue contains several of these same compounds, it also shows a surprising number of novel peaks in the NMR spectrum that have yet to be identified. Several of these small unidentified peaks between 1.5 and 1 ppm were found previously in one other orb-weaver species, *Zygiella atrica,* but others are completely novel [25,34] (electronic supplementary material). The spectral peaks for known LMMCs found in other orb-weavers were also relatively small in *Cyrtarachne* glue droplets suggesting that *C. akirai* glue is composed mostly of novel compounds whose identity could help to explain the unusual behaviour of the glue. Novel LMMCs may help to explain the hardening of the glue droplets over time and/or help to maintain cohesion of the glycoproteins without inhibiting the low viscosity of the droplets.

While the current investigation reveals insights into the performance of *C. akirai* glue compared to more typical orb-weaving spiders, the glue ultimately has to stick to a very different surface than the hydrophilic glass used in this current study—moth cuticles are both highly hydrophobic and intricately micro-structured. Future investigation should focus on the role of viscosity and droplet size for how aggregate glue spreads upon such surfaces. For instance, the low viscosity of *C. akirai* droplets might allow them to better spread within porous surfaces, such as moth scales, as low viscosity is often correlated with low surface tension which leads to higher capillary forces and spreading ability [9].

Further investigation of adhesion on three-dimensionally complex (similar to moth wings with scales) and hydrophobic surfaces is needed to better understand how these properties and chemistry interact to give *Cyrtarachne* glue high optimum strength on moths.

## Supplementary Material

Supplementary Tables and Figures

## Supplementary Material

Presented Data

## Data accessibility

Raw data used to create figures 2, 3, 4, 6, 7, and determine the percentage of salt components by mass are all available (as one Excel workbook) as the electronic supplementary material.
