# Supplementary material for: Supersaturation with water explains the unusual adhesion of aggregate glue in the webs of the moth-specialist spider, Cyrtarachne akirai
Source: R Soc Open Sci. 2018 Nov 7;5(11):181296. doi: 10.1098/rsos.181296 (PMC6281915; doi:10.1098/rsos.181296)
Supplement: Supplementary Tables and Figures [file rsos181296supp1.doc]

**Appendix**

| **Compound** | **Chemical Shift (nature of peak)** | **% Relative Composition** |
| --- | --- | --- |
| Betaine | 3.24(s), 3.87(s) | 11 |
| Choline | 3.18(s),3.49(m),4.03(m) | 5 |
| Glycine | 3.53(s) | 7 |
| NAT | 1.97(s), 3.06(t), 3.54(t) | 4 |
| NAP | 1.55(m),1.66(m),1.97(s),2.99(t),3.18(t) | 20 |
| Putrescine | 1.73(m),3.02(m) | 47 |
| Taurine | 3.23 (m),3.40(m) | 6 |

**Supplementary Table 1:** Table shows the chemical shifts (in ppm), nature of the peak detected [s: singlet, t: triplet and m: multiplet] and the % relative compositions of the various hygroscopic compounds found in the capture glue of *Cyrtarachne akirai*. (NAT: N-acetyl taurine, NAP: N- acetyl putrescine)

| Species | Optimum Humidity | Volume at Room Humidity (40 – 60 RH)  (um^3) | Work to Release (uJ) | Normalized Work to Release (uJ/um^3) |
| --- | --- | --- | --- | --- |
| *Verrucosa* | 50 | 281086 | 0.55 +/- .05 | 0.00617 +/- 0.000604 |
| *Larinioides* | 50 | 94253.95 | 1.20 | 0 |
| *Neoscona* | 70 | 317327.3 | 2.36 +/- .3 | 0.00254 +/- 0.000323 |
| *Tetragnatha* | 90 | 42700.69 | 0.15 +/- 0.02 | 0.0208 +/- 0.00328 |
| *Cyrtarachne* | 90 | 439749.7 | 9.46 +/- 3.61 | 0.00496 +/- 0.001.89 |
| *Cyrtarachne* (dry) | 90 | 832.07 | 0.29 ± 0.0074 |  |

**Supplementary Table 2:** Work to Release from glass for several orb-weaver species at their optimum humidity and work to release normalized by average volume of glue per um at room humidity.


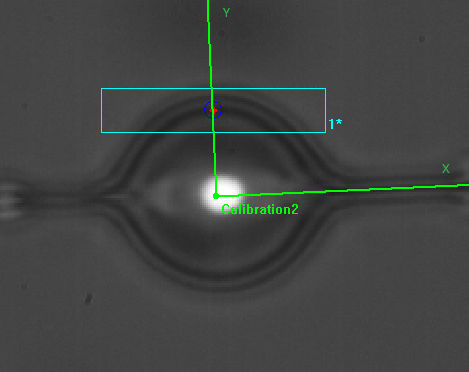


**Supplementary Fig. 1: Boundary tracking of a glue droplet during video analysis.** Each droplet was assigned its own coordinate system originating at the center . The +Y axis droplet edge was defined as the perpendicular of the thread axis (X) and then tracked across all frames of the video as shown above. These coordinates were then converted into radius and plotted over time.
